# Trends and Epidemiology of Fall-Related Hospitalizations Among Older Adults in the Split-Dalmatia County, Croatia: A Retrospective Descriptive Study from 2020 to 2024

**DOI:** 10.3390/medicina62071270

**Published:** 2026-06-30

**Authors:** Ivana Marasović Šušnjara, Gabriela Glavaš, Mladenka Parlov, Nora Josipa Savičević, Anamarija Jurčev Savičević, Hrvoje Šušnjara

**Affiliations:** 1 Faculty of Health Sciences, University of Split, 21000 Split, Croatia; 2 Teaching Institute for Public Health, Split-Dalmatia County, 21000 Split, Croatia; 3 University Hospital Split, 21000 Split, Croatia; 4 General Medicine Practice, 21000 Split, Croatia; 5 School of Medicine, University of Split, 21000 Split, Croatia

**Keywords:** falls, older adults, epidemiology, hospitalization, hip fracture, Croatia

## Abstract

*Background and Objectives*: Falls represent a major public health concern among older adults, contributing substantially to morbidity, mortality, and healthcare burden. This study aimed to analyze trends, demographic characteristics, hospitalization rates, injury patterns, and outcomes of fall-related hospitalizations among individuals aged ≥65 years in Split-Dalmatia County, Croatia, from 2020 to 2024. *Materials and Methods*: A retrospective epidemiological study was conducted using fall-related hospitalization data from the National Health Information System. Discharges for injury (S00-T98) caused by a fall (W00-W19) were selected using ICD-10. Analyses included individuals aged ≥65 years and were stratified by age group (65–74, 75–84, ≥85 years), sex, and year. Hospitalization rates per 100,000 population were calculated using official population data. Differences were assessed using chi-square and Kruskal–Wallis tests, while Poisson regression was used to estimate rate ratios (RRs) with 95% confidence intervals (CIs) and assess temporal trends. *Results:* A total of 4737 fall-related hospitalizations were recorded among individuals aged ≥65 years (58.32% of all cases). Hospitalization rates increased markedly with age, with individuals aged ≥85 years having more than a fivefold higher rate compared to those aged 65–74 years (RR = 5.16, 95% CI 4.80–5.56). Women accounted for 69.83% of cases and had higher hospitalization rates than men (RR = 1.74, 95% CI 1.64–1.85). Hip and femur injuries were the most common (50.39%). In-hospital mortality was 3.02% and higher among men. No significant temporal trend was observed (AAPC = −2.18%, *p* = 0.392). *Conclusions:* Fall-related hospitalizations are strongly associated with advanced age and female sex, with hip fractures predominating. Although rates remained stable over time, ongoing population ageing is likely to increase the future burden. Targeted, age- and sex-specific prevention strategies should be prioritized.

## 1. Introduction

Falls among older adults represent a major and growing public health challenge worldwide. With increasing life expectancy and rapid population ageing, the burden associated with falls is expected to rise substantially. According to the World Health Organization, falls are the second leading cause of injury-related mortality globally, accounting for approximately 684,000 deaths annually, while an estimated 37.3 million falls require medical attention [1]. Beyond acute physical injury, falls are strongly associated with functional decline, loss of independence, fear of recurrent falls, reduced quality of life, and increased healthcare utilization, thereby posing high societal and economic costs [1,2].

Population ageing is a key driver of the rising incidence of falls. Globally, the number of individuals aged ≥60 years is projected to increase markedly in the coming decades [3]. Europe is among the most rapidly ageing regions, with more than 20% of the population already aged 65 years or older [4]. Croatia follows similar demographic trends, including Split-Dalmatia County, where the proportion of older adults is steadily increasing [5]. These demographic shifts are expected to result in a parallel increase in age-related conditions, particularly falls and their associated complications.

Falls are defined as events in which an individual unintentionally comes to rest on the ground or a lower level [1] and are inherently multifactorial. Their occurrence reflects a complex interaction between intrinsic factors—such as age-related physiological changes, chronic diseases, cognitive impairment, and medication use—and extrinsic factors, including environmental hazards and unsafe living conditions [6,7,8]. The risk of falling increases cumulatively with the number of present risk factors [1].

Epidemiological data indicate that approximately one-third of individuals aged ≥65 years experience at least one fall annually [1], with incidence increasing with advancing age. Falls occur across various settings, including community, long-term care, and hospital environments, with consistently higher rates observed among institutionalized and hospitalized populations [7,9,10]. In Croatia, falls represent the leading external cause of injury-related hospitalizations, accounting for approximately 50% of such admissions [11], with hip fractures among the most frequent and severe consequences [11,12].

The impact of falls extends well beyond physical injury. Although most falls result in minor trauma, approximately 10–15% lead to fractures, and a smaller proportion to serious injuries such as traumatic brain injury [13,14]. Falls are a major cause of hospitalization, long-term disability, and mortality among older adults [15]. Moreover, they are associated with significant psychological sequelae, including fear of falling, reduced mobility, social isolation, and diminished quality of life [16,17]. Recurrent falls are common and substantially increase the risk of institutionalization and dependency [16].

Importantly, a considerable proportion of falls is preventable. It is estimated that 25–40% of falls can be avoided through targeted interventions [1]. Current evidence-based approaches emphasize multifactorial risk assessment followed by individualized, multicomponent interventions, including exercise programs, home hazard modification, medication review, and optimization of chronic disease management [1,9,18].

Despite the well-established global burden of falls, important gaps remain in the literature. Specifically, there is a lack of recent, region-specific epidemiological data from South-eastern Europe, including Croatia, that comprehensively capture temporal trends, demographic patterns, injury characteristics, and clinical outcomes of falls in older populations. Existing studies are often limited by narrow settings, short observation periods, or insufficient stratification by age and sex [6,10], which restricts their applicability for public health planning and targeted prevention strategies.

Given these limitations, robust population-based data are needed to better understand the local epidemiology of falls and inform context-specific interventions. Therefore, this study aims to analyze the incidence, demographic characteristics, injury types, hospitalization duration, and outcomes of falls among individuals aged ≥65 years in Split-Dalmatia County over five years (2020–2024), with particular emphasis on age- and sex-specific patterns.

## 2. Materials and Methods

### 2.1. Study Design, Setting and Data Sources

A retrospective descriptive epidemiological study was conducted to assess fall-related hospitalizations among individuals aged ≥65 years in Split-Dalmatia County, Croatia, between 2020 and 2024. Split-Dalmatia County is the second most populous county in Croatia, with 423,407 inhabitants according to the 2021 Population Census, representing approximately 10.9% of the national population. Like the rest of Croatia and many European regions, the country is experiencing pronounced population ageing, with the proportion of residents aged ≥65 years projected to increase from approximately 20% in 2020 to nearly 30% by 2050 [5]. Hospitalization data were obtained from the National Health Information System of the Croatian Institute of Public Health and included all residents of Split-Dalmatia County hospitalized at the University Hospital of Split, the main tertiary care center in the region. The University Hospital of Split is a publicly funded tertiary care institution within Croatia’s universal healthcare system, which is financed through mandatory social health insurance administered by the Croatian Health Insurance Fund, the country’s single purchaser of healthcare services [19].

Annual mid-year population estimates stratified by age group and sex were obtained from the Croatian Bureau of Statistics and used as denominators for the calculation of hospitalization rates. Detailed population data are provided in the Supplementary Material (Tables S1 and S2).

### 2.2. Study Population and Case Definition

The study population included all individuals aged ≥65 years residing in Split-Dalmatia County who were hospitalized due to falls during the study period.

Falls were identified using the International Classification of Diseases, 10th Revision (ICD-10) codes W00–W19. Injury types were classified using ICD-10 diagnostic codes (S00–S99) and grouped by anatomical region [20].

Each hospitalization was treated as a separate event. Transfers between hospital departments during the same episode of care were treated as a single hospitalization, whereas readmissions occurring after discharge were considered separate hospitalization events.

Participants were categorized into three age groups: 65–74, 75–84, and ≥85 years. Analyses were performed for the total population and stratified by age group, sex, and year of hospitalization.

### 2.3. Variables

The following variables were analyzed: number of hospitalizations due to falls, hospitalization rates, age, sex, year of hospitalization, type of injury, length of hospital stay (≤7 days, ≥8 days), and in-hospital mortality.

### 2.4. Statistical Analysis

Categorical variables were summarized using frequencies and percentages, while continuous variables were presented as medians and interquartile ranges (IQR). Normality of distribution was assessed using the Kolmogorov–Smirnov test.

Differences in categorical variables were evaluated using the chi-square (χ^2^) test or Fisher’s exact test when expected cell counts were <5. Differences in continuous variables were assessed using the Kruskal–Wallis test.

Hospitalization rates per 100,000 population were calculated using annual mid-year population estimates as denominators.

Poisson regression models with a log link function were used to estimate rate ratios (RR) with 95% confidence intervals (95% CI), using population size as an offset variable.

Temporal trends were assessed by including year as a continuous variable in the Poisson regression models. The average annual percent change (AAPC) was derived from the regression coefficient of the year variable.

A two-sided *p*-value < 0.05 was considered statistically significant. No adjustment for multiple comparisons was applied, except where explicitly stated.

All analyses were performed using STATISTICA version 13 (TIBCO Software Inc., Palo Alto, CA, USA).

The study is reported in accordance with the STROBE guidelines for observational studies (Table S3).

## 3. Results

### 3.1. Hospitalizations and Demographic Characteristics

From 2020 to 2024, a total of 8122 hospitalizations due to falls were recorded, of which 4737 (58.32%) occurred in individuals aged ≥65 years.

The distribution of hospitalizations among individuals aged ≥65 years differed significantly across age groups (χ^2^ test, *p* < 0.001), with a predominance of those aged 75–84 years. Notably, the proportion of patients aged ≥85 years increased over time, indicating a shift toward older age groups (Table 1). Among hospitalized patients aged ≥65 years, the median age ranged from 79 to 81 years, with no statistically significant differences between years (Kruskal–Wallis H = 4.78, *p* = 0.311).

A significantly lower proportion of male compared to female patients was observed across all years (χ^2^ test, *p* < 0.001). Overall, women accounted for 69.83% of hospitalizations (Table 2).

### 3.2. Hospitalization Rates

**Figure 1:**
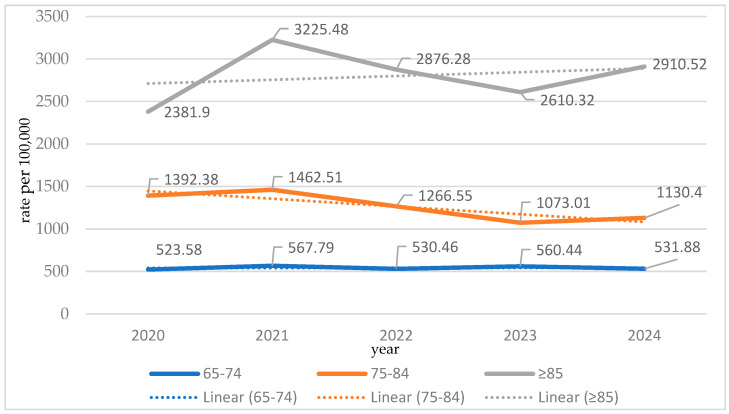
Hospitalization rates (per 100,000 population) by age group, 2020–2024.

**Figure 2:**
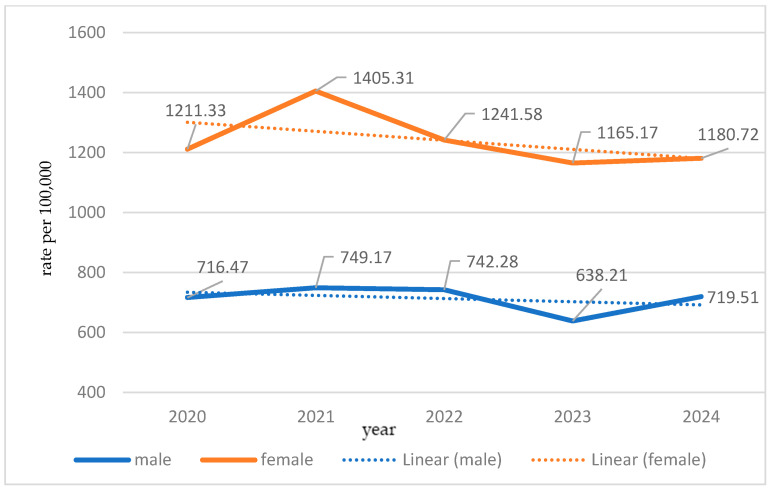
Hospitalization rates (per 100,000 population) by sex, 2020–2024.

Trends in hospitalization rates by age group and sex are presented in Figure 1 and Figure 2. Hospitalization rates increased markedly with advancing age, reaching the highest levels in individuals aged ≥85 years across all observed years (Figure 1).

Poisson regression analysis confirmed a strong age gradient in hospitalization rates. Compared with individuals aged 65–74 years, those aged 75–84 years had more than twice the hospitalization rate (RR = 2.32, 95% CI 2.17–2.49), while individuals aged ≥85 years had more than a fivefold higher rate (RR = 5.16, 95% CI 4.80–5.56).

Women had significantly higher hospitalization rates than men (RR = 1.74, 95% CI 1.64–1.85) (Figure 2).

Despite year-to-year variability, no significant temporal trend was observed (AAPC = −2.18%, *p* = 0.392), indicating overall stability in hospitalization rates during the study period.

### 3.3. Injury Types

As shown in Table 3, hip and thigh injuries clearly predominated, comprising over half of all fall-related hospitalizations (50.39%). These injuries were disproportionately more frequent among women, who accounted for more than three-quarters of cases.

Women also had a significantly higher proportion of injuries affecting the abdomen and pelvis, upper extremities, and lower legs (all *p* < 0.001). In contrast, injuries to the head, neck, thorax, and ankle/foot showed no significant sex-related differences.

### 3.4. Length of Hospital Stay

The distribution of hospital stay duration differed across years (Table 4). Overall, hospitalizations lasting ≥8 days were slightly more frequent (51.99%). A statistically significant difference across years was observed (χ^2^ test, *p* = 0.006). Although longer hospitalizations (≥8 days) were slightly more frequent overall, a shift toward shorter hospital stays was observed in later years.

### 3.5. In-Hospital Mortality

In-hospital mortality ranged from 2.1% to 4.0%, with an overall rate of 3.02% (Table 5).

Despite the predominance of female patients among hospitalizations, mortality was consistently higher in men across most years, with statistically significant sex differences observed (χ^2^ test, *p* < 0.05). This pattern was evident throughout the study period, although the difference was not significant in 2024.

Overall, these findings indicate that fall-related hospitalizations in older adults are characterized by a high burden of hip and thigh injuries, prolonged hospital stays, and non-negligible in-hospital mortality, particularly among men.

## 4. Discussion

This population-based study analyzes fall-related hospitalizations among people aged 65 and older in Split-Dalmatia County in the period 2020–2024. Key findings include a marked increase in hospitalization rates related to age, a higher female representation, and a predominance of hip and femur fractures. Hospitalization duration was also assessed as part of the overall burden of fall-related admissions. Although the population is undergoing demographic ageing, no statistically significant linear temporal trend in hospitalization rates was identified over the five-year study period. This result should be interpreted in the context of methodological limitations, including the relatively short observation period, which may be insufficient to capture longer-term trends, and the assessment of only linear changes, which may overlook non-linear patterns.

In addition, an apparent increase in hospitalization rates observed in 2021 may reflect disruptions in healthcare utilization during the COVID-19 pandemic, potentially affecting admission patterns and obscuring underlying temporal dynamics [21].

Therefore, the absence of a significant linear trend should not be interpreted as evidence of stable incidence of fall-related hospitalizations, but rather as a possible reflection of short-term fluctuations, pandemic-related effects, and/or relatively stable hospitalization practices within the observed period.

Because previous Croatian studies have largely focused on individual hospitals or specific injury types, this study provides one of the first population-based estimates of fall-related hospitalization rates among older adults in Split-Dalmatia County [21,22,23]. Although direct international comparisons should be interpreted with caution due to differences in healthcare system organization, admission criteria, and case definitions, the observed hospitalization patterns are broadly consistent with those reported in several European countries, where rates increase steeply with advancing age, particularly among individuals aged ≥85 years [1,24,25].

Nevertheless, the regional data also highlight important local priorities for prevention. The pronounced increase in hospitalization rates among the oldest age groups and the predominance of hip fractures support the need for targeted community-based fall prevention strategies, improved osteoporosis management, and strengthened multidisciplinary geriatric care, particularly for the oldest segment of the population [23,26].

The age-dependent hospitalization increase is consistent with existing evidence [1,27,28,29,30], with the oldest age groups having a multi-fold higher risk. Although the effect shown by our study is larger compared to some earlier estimates, the finding is biologically and clinically plausible considering the accumulation of frailty and multi-morbidity [31,32]. At the same time, the selection of more severe cases inherent in the hospitalization data should be taken into account, which is why the results primarily reflect more serious outcomes and not the overall average population risk of falls.

Women accounted for the majority of hospitalizations and higher admission rates, consistent with previous domestic [11] and international [27,33,34,35,36] research findings. These differences likely resulted from the interaction of biological factors (e.g., osteoporosis), demographic patterns (longer life expectancy), and differences in risk exposure [30,37,38]. Because variables such as comorbidities and functional status were not available, it was not possible to fully control the confounding factors; the findings should be interpreted descriptively rather than causally.

Although the incidence of women’s hospitalizations was higher, men had higher in-hospital mortality. This finding is consistent with the previous studies [8,39,40], but requires a cautious interpretation. Possible explanations include the differences in injury severity, comorbidities, and health care utilization patterns [41]. It is also possible that hospitalized men represent a selected group with a more severe clinical profile.

The dominance of hip and femur fractures among hospital admissions reflects the nature of the data source used, which inherently includes more severe injuries. Milder cases are likely to remain outside the hospital registers. A similar pattern has been reported in other hospital-based studies [42], while the community-based studies have shown a wider range of injuries [27,41,43]. Therefore, these results do not reflect the distribution of all falls, but rather those that have been clinically treated. Hip fractures are associated with high morbidity and mortality, frequent surgical treatment, and long-term functional limitations, with a significant proportion of patients not returning to their previous level of mobility [42]. Consequently, this leads to longer hospital treatments, the need for rehabilitation, and increased healthcare costs.

The absence of a significant age-related trend may indicate a stabilization of risk, but also changes in the organization of the health system. The impact of the COVID-19 pandemic, which has changed the patterns of health care utilization and the hospital capacity availability, should be considered in particular [28]. Without primary care and community data, it is not possible to distinguish whether these are real epidemiological changes or changes in the health system.

Shortening the length of hospitalisation may reflect the increased care efficiency [44], but also system reorganization, including earlier discharge and transfer of care toward nonhospital or social elements [44]. Therefore, this indicator should be interpreted as an indicator of hospital practice, not the total duration of treatment.

### 4.1. Strengths and Limitations

The main strength of this study lies in its population-based design and the use of standardized administrative data collected consistently over a five-year period, ensuring comprehensive coverage of hospitalizations for fall-related injuries in the study region.

Several limitations should be acknowledged. First, the use of hospitalization data introduces a selection bias toward more severe cases and may be affected by potential coding inaccuracies inherent to administrative databases. In addition, the lack of individual-level clinical information, including comorbidities, medication use, functional status, environmental circumstances of the fall, and place of residence (community versus long-term care facility), limited the ability to examine determinants of falls and to perform detailed subgroup analyses.

Furthermore, outcome assessment was restricted to in-hospital mortality, as no post-discharge follow-up data were available. As a result, the long-term consequences of falls, including recurrent falls, functional decline, institutionalization, and long-term mortality, could not be evaluated. Finally, although the study provides region-specific data, generalizability to other settings may be limited due to differences in healthcare systems, population structure, and care pathways.

### 4.2. Clinical Implications

Despite the limitations, the results have clear clinical implications. The high incidence of hip and femur fractures highlights the need for integrated strategies that include fall prevention and osteoporosis treatment [1,45], in line with recent international guidelines. Also, the higher mortality among men suggests potential for targeted interventions, although further research is needed to clarify the causes.

## 5. Conclusions

This population-based study provides a comprehensive epidemiological overview of fall-related hospitalizations among adults aged ≥65 years in Split-Dalmatia County over a five-year period (2020–2024). The findings demonstrate a marked increase in hospitalization rates with age, a predominance of female patients, hip and femur fractures as the leading injury types, and higher in-hospital mortality among men. Hospitalization duration was also assessed as part of the overall burden of fall-related admissions.

Although no significant linear temporal trend was observed during the study period, this finding should be interpreted with caution due to the relatively short observation window and the potential influence of the COVID-19 pandemic on hospitalization patterns.

Overall, these results are consistent with international evidence and provide relevant regional epidemiological data to inform targeted fall prevention strategies and geriatric care planning in older adults. Future research should incorporate individual-level risk factors, residential setting, and longitudinal follow-up to enable a more comprehensive understanding of the determinants and long-term outcomes of fall-related injuries among older adults.

## Figures and Tables

**Table 1:** Distribution of fall-related hospitalizations by age group and year.

Year	65–74 *n* (%)	75–84 *n* (%)	≥85 *n* (%)	Total *n*	χ^2^ *	*p*
2020	275 (30.32)	393 (43.33)	239 (26.35)	907	42.93	<0.001
2021	309 (29.91)	402 (38.92)	322 (31.17)	1033	14.73	0.001
2022	289 (30.29)	373 (39.10)	292 (30.61)	954	14.28	0.001
2023	309 (34.72)	316 (35.51)	265 (29.78)	890	5.15	0.076
2024	296 (31.06)	347 (36.41)	310 (32.53)	953	4.37	0.112
**Total**	**1478 (31.20)**	**1831 (38.65)**	**1428 (30.15)**	**4737**	**61.12**	**<0.001**

* The χ^2^ test was used to assess differences in age distribution across years.

**Table 2:** Distribution of fall-related hospitalizations by sex and year.

Year	Male *n* (%)	Female *n* (%)	Total (*n*)	χ^2^ *	*p* Value
2020	279 (30.76)	628 (69.24)	907	134.29	<0.001
2021	295 (28.56)	738 (71.44)	1033	189.98	<0.001
2022	296 (31.03)	658 (68.97)	954	137.63	<0.001
2023	259 (29.10)	631 (70.90)	890	155.49	<0.001
2024	300 (31.48)	653 (68.52)	953	130.75	<0.001
**Total**	**1429 (30.17)**	**3308 (69.83)**	**4737**	**745.33**	**<0.001**

* The χ^2^ test was used to assess differences in age distribution across years.

**Table 3:** Distribution of injury types among hospitalized patients due to falls by sex, Split-Dalmatia County, 2020–2024.

ICD-10 *	Injury Type	Total *n* (%)	Male *n* (%)	Female *n* (%)	*p*-Value
S00–S09	Head	558 (11.78)	273 (48.92)	285 (51.08)	0.611
S10–S19	Neck	38 (0.8)	24 (63.16)	14 (36.84)	0.105
S20–S29	Thorax	520 (10.98)	248 (47.69)	272 (52.31)	0.293
S30–S39	Abdomen and pelvis	479 (10.11)	146 (30.48)	333 (69.52)	<0.001
S40–S49	Shoulder and upper arm	165 (3.48)	46 (27.88)	119 (72.12)	<0.001
S50–S59	Elbow and forearm	134 (2.83)	30 (22.39)	104 (77.61)	<0.001
S60–S69	Wrist and hand	8 (0.17)	6 (75.0)	2 (25.0)	—
S70–S79	Hip and thigh	2387 (50.39)	533 (22.33)	1854 (77.67)	<0.001
S80–S89	Knee and lower leg	332 (7.0)	81 (24.4)	251 (75.6)	<0.001
S90–S99	Ankle and foot	26 (0.55)	16 (61.54)	10 (38.46)	0.239

* Abbreviations: ICD-10, International Classification of Diseases, 10th Revision. *p*-values were calculated using the chi-square test; Fisher’s exact test was applied when expected cell counts were <5. Categories with very small counts were not tested. A Bonferroni correction was applied for multiple comparisons.

**Table 4:** Length of hospital stay due to falls by year, Split-Dalmatia County, 2020–2024.

Year	≤7 Days *n* (%)	≥8 Days *n* (%)	Total (*n*)	*p*-Value *
2020	414 (45.64)	493 (54.36)	907	0.009
2021	447 (43.27)	586 (56.73)	1033	<0.001
2022	437 (45.81)	517 (54.19)	954	0.010
2023	457 (51.35)	433 (48.65)	890	0.421
2024	519 (54.46)	434 (45.54)	953	0.006
**Total**	**2274 (48.01)**	**2463 (51.99)**	**4737**	**0.006**

* Chi-square test was used to compare the distribution of hospital stay duration (≤7 vs. ≥8 days) across years.

**Table 5:** In-hospital mortality due to falls by sex and year, Split-Dalmatia County, 2020–2024.

Year	Sex	Alive *n* (%)	Death *n* (%)	Total *n*	*p*-Value *
2020	Total	878 (96.80)	29 (3.20)	907	0.004
	Male	263 (94.27)	16 (5.73)	279	
	Female	615 (97.93)	13 (2.07)	628	
2021	Total	992 (96.03)	41 (3.97)	1033	0.010
	Male	276 (93.56)	19 (6.44)	295	
	Female	716 (97.02)	22 (2.98)	738	
2022	Total	929 (97.38)	25 (2.62)	954	<0.001
	Male	280 (94.59)	16 (5.41)	296	
	Female	649 (98.63)	9 (1.37)	658	
2023	Total	862 (96.85)	28 (3.15)	890	0.013
	Male	245 (94.59)	14 (5.41)	259	
	Female	617 (97.78)	14 (2.22)	631	
2024	Total	933 (97.90)	20 (2.10)	953	0.734
	Male	293 (97.67)	7 (2.33)	300	
	Female	640 (98.01)	13 (1.99)	653	
**Total**		**4594 (96.98)**	**143 (3.02)**	**4737**	

* Chi-square test was used to compare mortality between sexes within each year.

## Data Availability

The data used in this study are available from the Croatian Institute of Public Health, but restrictions apply to their availability.

## Supplementary Materials

The following supporting information can be downloaded at: https://www.mdpi.com/article/10.3390/medicina62071270/s1, Table S1: Hospitalization rates (per 100,000 population) by age group, 2020–2024. Table S2: Hospitalization rates (per 100,000 population) by sex, 2020–2024. Table S3: STROBE Statement—Checklist of items that should be included in reports of cross-sectional studies.

## Abbreviations

The following abbreviations are used in this manuscript:

AAPC	Average Annual Percent Change
CI	Confidence Interval
ICD-10	International Classification of Diseases, 10th Revision
IQR	Interquartile Range
RR	Rate Ratio
STROBE	STrengthening the Reporting of OBservational studies in Epidemiology
